# Electrical storm after correction of an uncomplicated congenital atrial septal defect in an adult: a case report

**DOI:** 10.1186/s12872-021-02164-6

**Published:** 2021-07-22

**Authors:** Ying Liang, Feilong Hei, Yulong Guan

**Affiliations:** Department of Extracorporeal Circulation, Fuwai Hospital, Chinese Academy of Medical Sciences and Peking Union Medical College, 167 Beilishi Road, Xicheng District, Beijing, 100037 People’s Republic of China

**Keywords:** Electrical storm, Ventricular arrhythmia, Congenital heart disease, Tricuspid valvuloplasty, Case report

## Abstract

**Background:**

There is a paucity of published literature describing electrical storm after the correction of uncomplicated atrial septal defect (ASD) in an adult.

**Case presentation:**

We present a 49-year-old woman with a congenital ASD combined with mild tricuspid regurgitation who denied any history of arrhythmia or other medical history. She suffered from electrical storm (≥ 3 episodes of ventricular tachycardias or ventricular fibrillations) in the early stage after ASD repair with combined tricuspid valvuloplasty. During electrical storm, her electrolytes were within normal ranges and no ischemic electrocardiographic changes were detected, which suggested that retained air embolism or acute coronary thrombosis were unlikely. Additionally, echocardiographic findings and her central venous pressure (5–8 mmHg during the interval between attacks) failed to support the diagnosis of pericardial tamponade. After a thorough discussion, the surgeons conducted an emergent re-exploration and repeated closure of the ASD with combined DeVega's annuloplasty. Eventually, the patient recovered uneventfully, without reoccurring arrhythmias during follow-up. Although we fail to determine the definite cause, we speculate that the causes probably are iatrogenic injury of the conduction system due to a rare anatomic variation, poor intraoperative protection, latent coronary distortion during tricuspid valvuloplasty, or idiopathic or secondary abnormalities of the conduction system.

**Conclusions:**

For most surgeons, performing re-exploration without a known etiology is a difficult decision to make. This case illustrates that re-exploration could be an option when electrical storm occurs in the early stage postoperatively. Nevertheless, surgeons should assess the benefit-risk ratio when taking this unconventional measure.

## Background

Atrial septal defect (ASD) is the third most common type of congenital heart disease and can present at any age [1]. This type of defect accounts for 25–30% of congenital heart defects diagnosed in adults [2]. In 2010, the European Society of Cardiology (ESC) recommended that adult patients with significant shunts (signs of right ventricular volume overload) and pulmonary vascular resistance < 5 Wood units should undergo ASD closure regardless of their symptoms (class I, level B) [3]. Moreover, recent studies have observed improvements in symptoms and exercise capacities even when ASD closures are conducted in elderly patients [4–6]. Severe complications after corrective surgery for ASD include bleeding, pneumothorax, and pericardial and pleural effusions, and for older patients, stroke, heart failure, left atrial hypertension and pulmonary venous congestion are also seen incidentally [1, 4]. Postoperative arrhythmias usually comprise transient atrial-ventricular block, atrial fibrillation or atrial flutter, supraventricular arrhythmia, and bradyarrhythmia [1, 4, 7, 8]. It is rare to encounter electrical storm, which is defined as the occurrence of 3 or more distinct episodes of ventricular tachycardia (VT) or ventricular fibrillation (VF) within 24 h (also known as VT/VF storm)[9]. In this case report, we present a 49-year-old woman without an arrhythmia history who experienced VT/VF storm after ASD repair with combined tricuspid valvuloplasty. Her symptoms improved after a repeated correction. We attempt to explore the underlying causes and treatments for this VT/VF storm.

## Case presentation

**Fig. 1:**
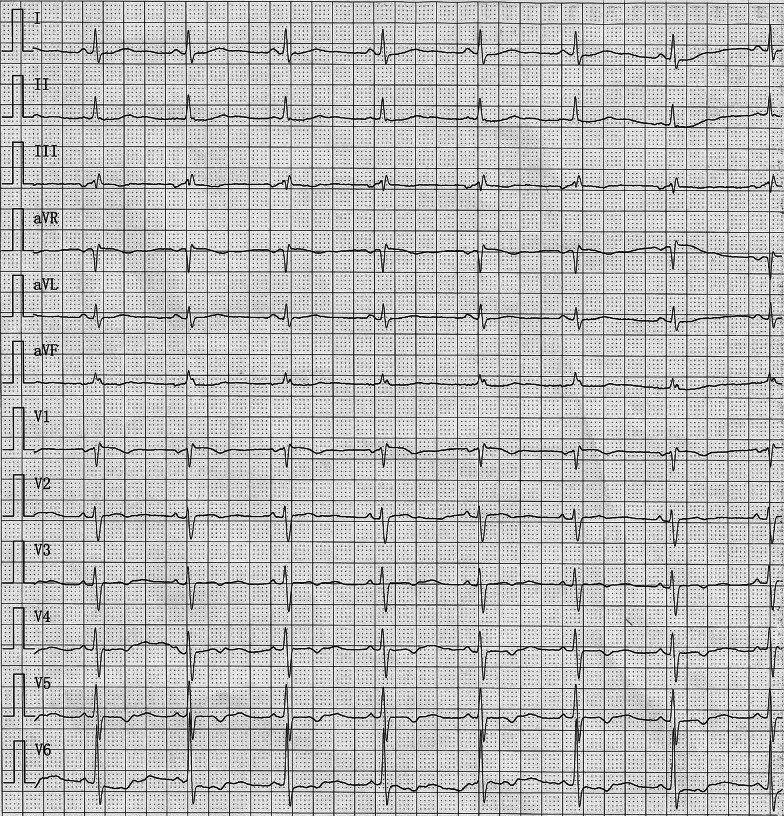
Preoperative 12-lead electrocardiogram

A 49-year-old woman complaining of exercise intolerance for 10 years was admitted to our center. Her echocardiography presented mild tricuspid regurgitation and a congenital ASD with a significant left-to-right shunt that caused structural enlargement of the right heart (echocardiographic data: LV: 41 mm; LA: 38 mm; RV: 24 mm; RA: 54 × 40 mm; EF: 60%). She denied any previous arrhythmia events or other past medical history. On admission, her preoperative 12-lead electrocardiogram (ECG) revealed sinus rhythm with an incomplete right bundle branch block (Fig. 1). Apart from grade 2 heart murmurs, her physical examinations were otherwise normal. Initial laboratory test results revealed normal levels of N-terminal pro-brain natriuretic peptide (NTproBNP), magnesium, and potassium. Moreover, her coagulation, biochemical, and hematological indices remained within the normal range. The patient presented as New York Heart Association (NYHA) functional class II, albeit with moderate lesions in the proximal segment of the left anterior descending artery confirmed by coronary computerized tomography (CT).

The patient underwent ASD repair with combined tricuspid valvuloplasty. After a median sternotomy, the surgeons placed an aortic cannula in the ascending aorta with bicaval cannulation to establish cardiopulmonary bypass (CPB). Under aortic cross-clamp conditions, cardiac arrest was achieved with antegrade cold blood cardioplegia. Then, CPB was instituted under mild hypothermia (34 °C). After opening the right atrium, a superior sinus venosus ASD with a size of 2.0 × 2.5 cm was identified. Then, the surgeons fixed the defect with running 5–0 Prolene sutures. Meanwhile, they carried out a concomitant DeVega's annuloplasty for tricuspid regurgitation. Ultimately, she was weaned from CPB uneventfully, with an overall duration of CPB of 63 min and a cross-clamp time of 17 min. Of note, two transient episodes of ventricular arrhythmias (VAs) occurred at the end of surgery. However, as sinus rhythm was restored promptly, the patient was transferred to the intensive care unit (ICU) while intubated for further observation. At the first admission to the ICU, her blood pressure remained within the normal range (102/63 mmHg), and the electrocardiogram revealed sinus rhythm.

**Fig. 2:**
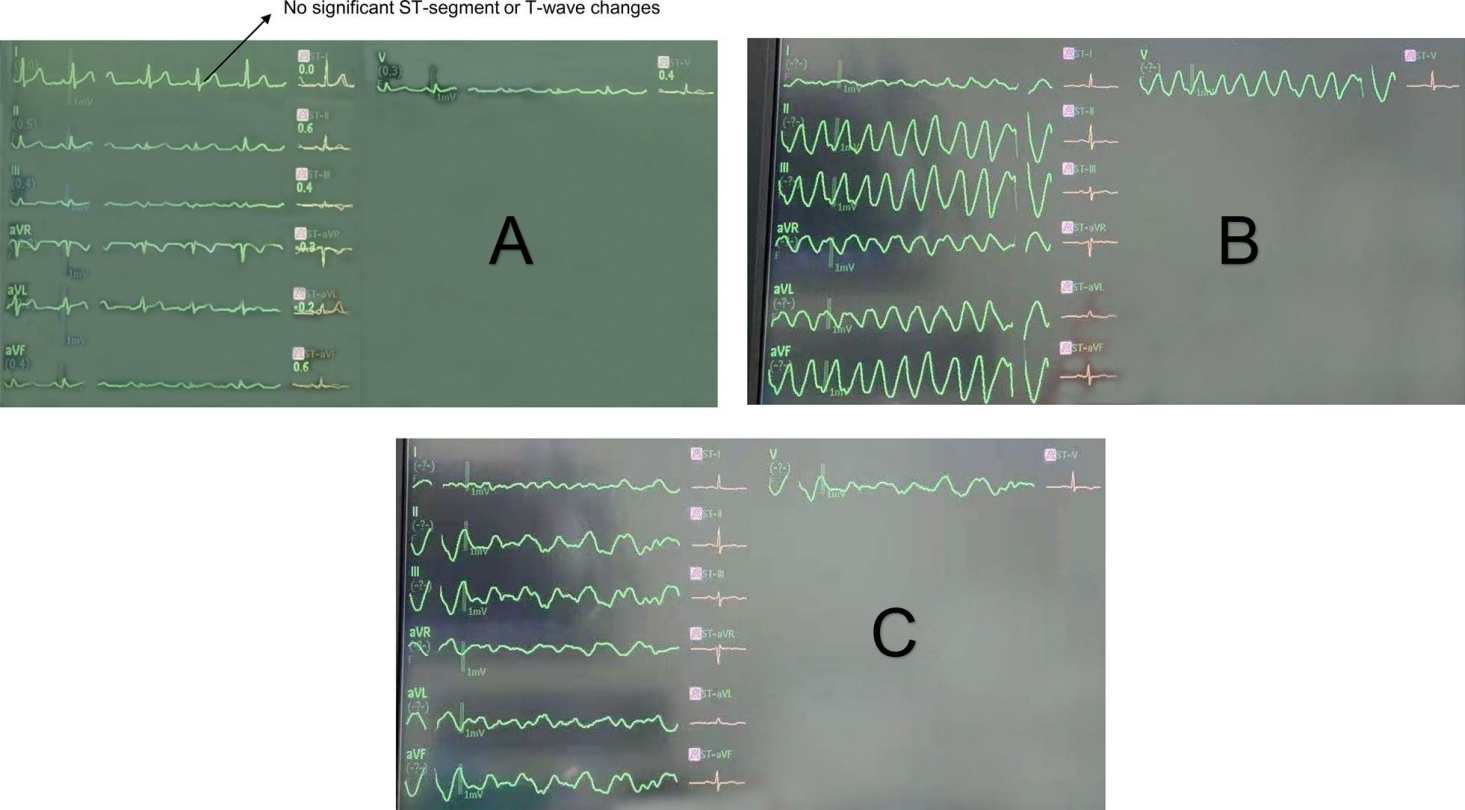
Electrocardiogram of electrical storm. **a** normal electrocardiogram during the interval between attacks; **b** ventricular tachycardias; **c** ventricular fibrillations

**Fig. 3:**
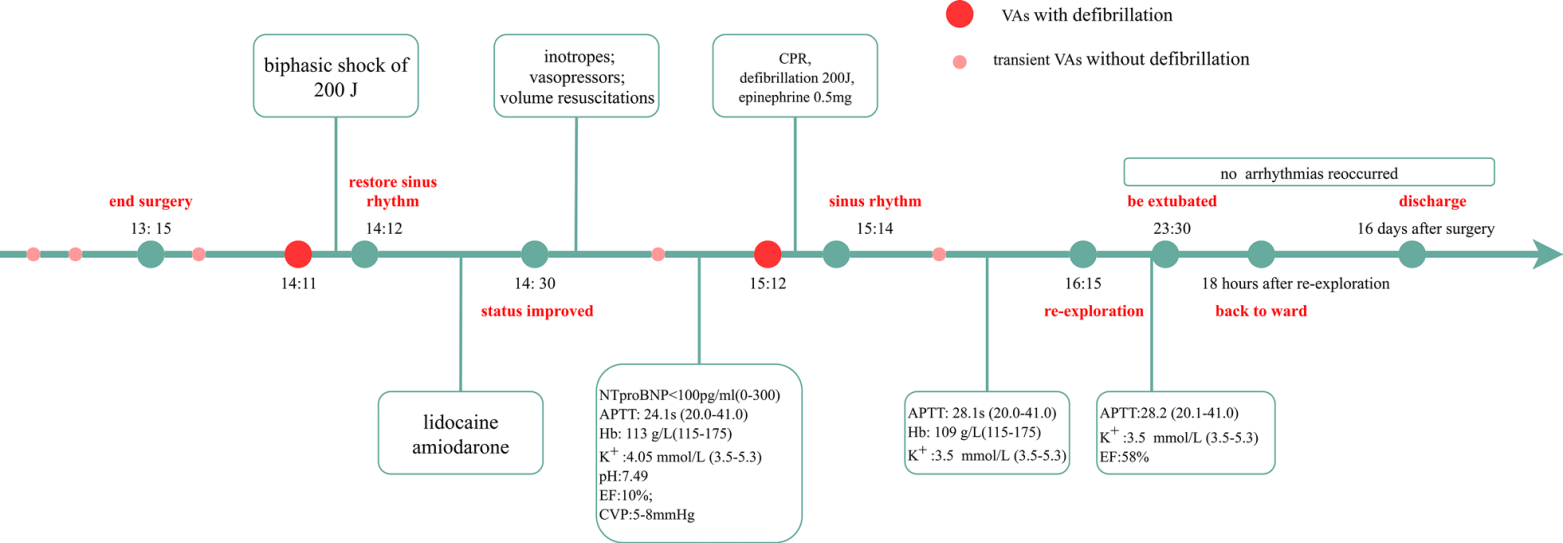
Timeline of the whole event. *APTT* activated partial thromboplastin time, *CPR* cardiopulmonary resuscitation, *CVP* central venous pressure, *EF* ejection fraction, *Hb* hemoglobin, *NTproBNP* N-terminal pro-brain natriuretic peptide, *VAs* ventricular arrhythmias

However, unanticipated VTs and sequent VFs occurred one hour after admission (Fig. 2b, c). Then, the ICU staff applied external defibrillation with a 200-J biphasic shock for prompt resuscitation and added antiarrhythmic medications (including amiodarone and lidocaine) to migrate subsequent premature ventricular contractions. Soon after, the patient recovered sinus rhythm. However, after an initial uneventful recovery, the patient frequently experienced recurrent episodes of VT/VFs. The whole event is shown in Fig. 3.

The heart team suspected four possible etiologies at the first episode: electrolyte disturbances, pericardial tamponade, retained air embolism or thrombosis in coronary arteries. However, point-of-care testing revealed the standard range of potassium. Although her EF was 10% when the first severe VT/VF storm occurred, the echocardiographic findings failed to show evidence of pericardial tamponade (no diastolic right ventricular collapse or pericardial effusion). Moreover, the central venous pressure (CVP) remained at 5–8 mmHg, despite a temporary elevation during the VT/VF storm. There were no significant ST-segment or T-wave changes during the interval between attacks and at the end of surgery (Fig. 2a). These results implied that coronary ischemia caused by coronary thrombosis or intracoronary air embolism was unlikely.

As VT/VF storm frequently transpired in the early postoperative stage and the transient arrhythmias also occurred before admission to the ICU, the cardiac experts hypothesized that the severe VA episodes might be associated with the surgical intervention. They attempted to explore the definite causes of this issue and focused on the two most likely explanations: trauma from the surgical sutures or underlying stenosis or occlusion of the right coronary artery caused by tricuspid valvuloplasty. Given the greater value of re-exploration in this emergent situation, we chose to forgo coronary angiography since it might not be able to detect the underlying coronary problem. After a thorough discussion, the surgeons decided to conduct an emergent re-exploration in case more life-threatening events occurred. Three hours after the first surgery, a senior surgeon initiated the re-exploration. Although no significant stitching errors were detected during the second surgery, the senior surgeon carefully replaced all sutures with slight adjustments and fixed the ASD and tricuspid leaflets again to address the two possible causes simultaneously. Ultimately, the patient was weaned from CPB, with a duration of 49 min and a cross-clamp time of 18 min.

After re-exploration, the patient’s clinical status improved, and no arrhythmias occurred. The EF was restored immediately when her status improved. Furthermore, the results of various examinations kept within normal limits before discharge. We asked cardiac electrophysiologists to help with ECG in the whole event. They agreed that no underlying signs of inherited arrhythmias were detected in the preoperative ECG, and it’s risky to conduct electrophysiological examination in that situation. As the patient displayed normal ECG over the 24 h after surgery, there was no indications for further electrophysiological examination postoperatively. Accordingly, we failed to obtain the electrophysiological study. During the 6-month follow-up, her recovery remained uneventful, without any episodes of arrhythmias.

## Discussion and conclusions

Although atrial flutters and atrial fibrillations are common in older patients with sizeable ASDs [1], VT/VF storm after the correction of ASD is rare. It is often an incidental finding after correction of tetralogy of Fallot (TOF) and left ventricular outflow tract defects [10, 11]. To the best of our knowledge, this is the first case in the literature.

**Fig. 4:**
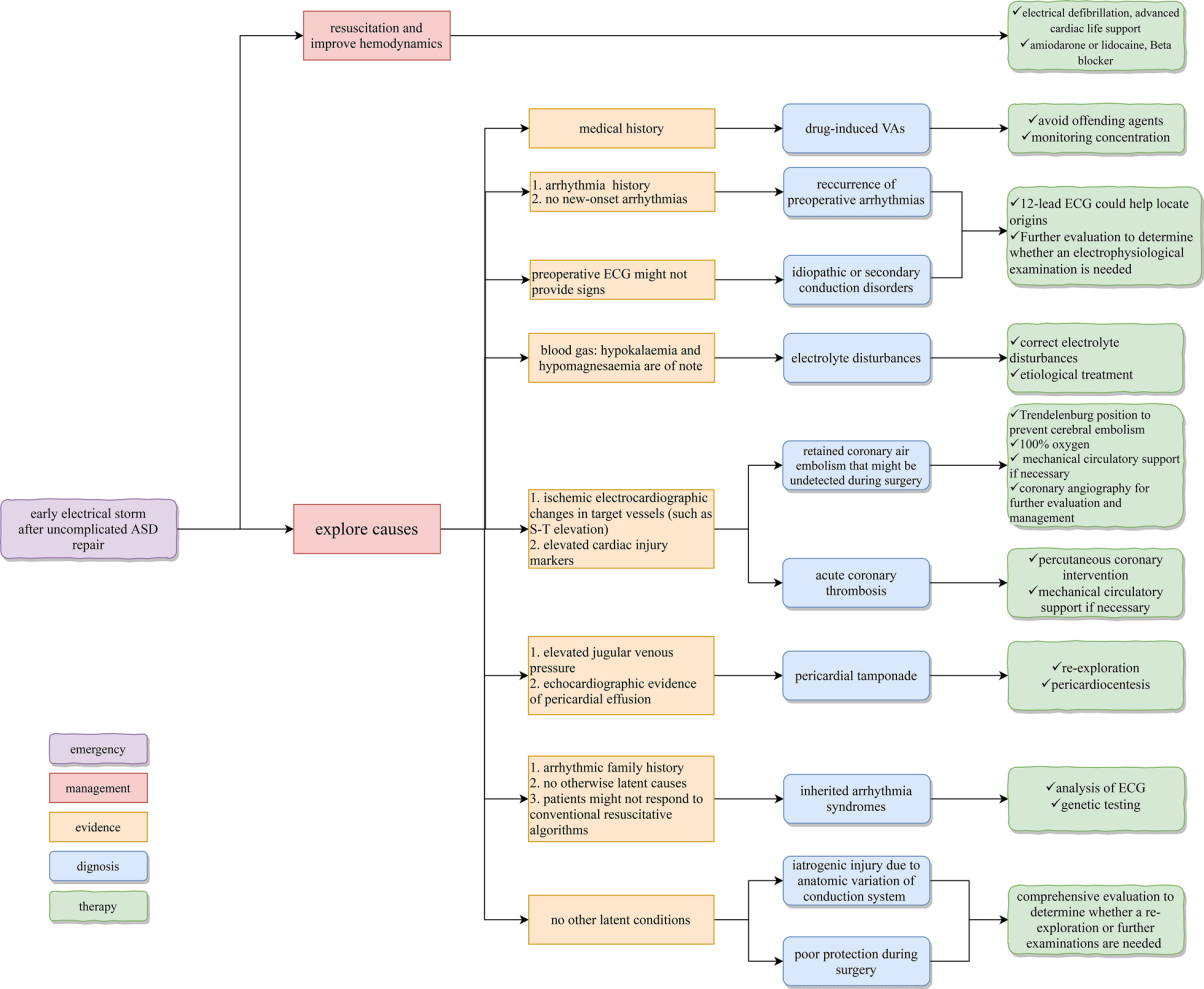
Treatment strategies when electrical storm occurs after uncomplicated ASD repair. *ASD* atrial septal defect, *ECG* electrocardiogram, *VAs* ventricular arrhythmias

Postoperative VAs are associated with increased mortality [12–14]. The underlying risk factors for electrical storm are shown in Table 1. The precise mechanism of VAs remains debatable. Underlying mechanisms involve the following: (1) abnormal or enhanced automaticity in ventricular myocytes and Purkinje fibers; (2) triggered activity induced by early or late afterdepolarizations; (3) reentry around a scar and functional block; and (4) reentry due to heterogeneity of ventricular repolarization [15]. Knowing the modifiable risk factors, identifying underlying causes, and providing effective management will improve patient prognosis when VAs occur. Based on our experience and previous literature, we have summarized the potential causes and treatment strategies when unexpected VT/VF storm occurs early after uncomplicated ASD repair in Fig. 4 [9, 15–17].

**Table 1:** Factors related to electrical storm in cardiac surgery

Advanced age [12, 15, 18]	Risky
Higher EF [12, 19, 20]	Protective
Peripheral vascular disease [12]	Risky
Off-pump surgery [12]	Protective
The need for emergent surgery [12]	Risky
Prolonged pump time [19]	Risky
Female [14, 19]	Risky
Pulmonary hypertension [19]	Risky
Systemic hypertension [19]	Risky
Medication-induced or congenital long QT syndrome [15, 20–22]	Risky
More complex surgery [18, 23]	Risky
Electrolyte disorders [18, 24]	Risky
Preoperative ventricular arrhythmia [20]	Risky
Iotropes required [14]	Risky

*EF* ejection fraction

The heart team decide to perform repeat exploration based on two hypotheses: trauma from the surgical sutures or underlying stenosis or occlusion of the right coronary artery caused by tricuspid valvuloplasty, and the two problems were managed simultaneously during the second surgery. The fact that VAs haven’t reoccurred after re-exploration confirms our assumption to some extent. Nevertheless, in the clinical setting, neither of the two causes could be completely confirmed, as coronary stenosis and conduction system damage could not be diagnosed during surgery. Additionally, Kojima reported a patient with a nontypical ECG of prolonged QT intervals who experienced refractory VTs during ASD surgery [21]. As the patient disclosed normal preoperative and postoperative ECG, meanwhile without related family history or medical history, we failed to perform genetic testing to confirm any inherited arrhythmia syndromes. Moreover, uncommon anatomic variations and idiopathic or secondary abnormalities of the conduction system could not be ruled out completely [25, 26]. As a whole, there is no definite evidence to date to locate the exact etiology of this VT/VF storm. Future efforts should focus on imaging methods that provide an accurate depiction of the morphology and function of the conduction system. These findings will be instructive for improving arrhythmic complications during cardiac surgery.

When VT/VF storm occurs in the early stage after uncomplicated ASD repair, the treatment strategies should focus on electrolyte disturbances, pericardial tamponade, retained air embolism or acute coronary thrombosis. Additionally, idiopathic or secondary abnormalities of the conduction system, inherited arrhythmia syndromes, iatrogenic injury of the conduction system due to anatomic variations or poor intraoperative protection should also be considered.

For most surgeons, performing re-exploration without a known etiology is a difficult decision to make. This case illustrates that re-exploration could be an option when emergent VT/VF storm occurs in the early postoperative stage. Nevertheless, surgeons should assess the benefit-risk ratio when taking this unconventional measure.

## Data Availability

Data sharing not applicable to this article as no datasets were generated or analyzed during the current study.

## Abbreviations

EF: Ejection fraction
LA: Left atrium
LV: Left ventricle
RA: Right atrium
RV: Right ventricle
